# Patient Presentations in a Community Pain Clinic after COVID-19 Infection or Vaccination: A Case-Series Approach

**DOI:** 10.3390/clinpract13060139

**Published:** 2023-12-11

**Authors:** Angela Mailis, Naomi Kupferstein, Demetry Assimakopoulos, Alex C. Mailis, Sean Sutton, Shehnaz Fatima Lakha

**Affiliations:** 1 Pain & Wellness Center, Vaughan, ON L6A 3Z3, Canada; naomi@thepwc.ca (N.K.); demetry@thepwc.ca (D.A.); alex.mailis@thepwc.ca (A.C.M.); ssutton@thepwc.ca (S.S.); sfatima.lakha@utoronto.ca (S.F.L.); 2 Department of Medicine, Division of Physical Medicine and Rehabilitation, University of Toronto, Toronto, ON M5S 3H2, Canada; 3 Comprehensive Integrated Pain Program, Toronto Rehabilitation Institute, Toronto, ON M5G 2A2, Canada; 4 Institute of Medical Sciences, Temerty Faculty of Medicine, University of Toronto, ON M5S 1A8, Canada

**Keywords:** community pain clinic, COVID-19 infection, vaccination

## Abstract

Objectives: Early case report studies and anecdotes from patients, medical colleagues, and social media suggest that patients may present to chronic pain clinics with a number of complaints post COVID-19 infection or vaccination. The aim of this study is to systematically report on a consecutive series of chronic pain patients seen in a community-based pain clinic, who acquired symptoms after COVID-19 infection or vaccination. Methods: This retrospective cross-sectional descriptive study identified all patients seen at the clinic over a 4-month period (January–April 2022) with persistent symptoms after COVID-19 infection, vaccination, or both. Information was collected on sex, gender, age, details of vaccination, new pains, or exacerbation of old pain plus the development of novel symptoms. Results: The study identified 21 community dwellers (17 females and 4 males; F/M 4.25/1; age range 22–79 years; mean age 46.3 years), with symptoms attributed to COVID-19 infection or vaccination. Several patients suffered exacerbation of previous pains or developed novel pains, as well as high levels of anxiety and mood disorders. A review of the existing literature provides support for the spectrum of symptoms displayed by the study group. Conclusions: Information collected in this study will add to the body of COVID-19-related literature and assist particularly community practitioners in recognizing and managing these conditions.

## 1. Background and Aims

The COVID-19 virus is the etiological agent of a novel infectious disease which has generated distressing effects worldwide of unimaginable proportions [1]. Just like most other medical specialties, the field of chronic pain is one of the hardest hit by the pandemic, leaving many patients overburdened with their chronic pain and their ongoing treatment [2].

The association of acute pain in the context of COVID-19 infection is well recognized. During acute infection, generalized myalgia and headache alongside upper respiratory symptoms were noted to be common symptoms [3].

Long term symptoms after COVID-19 infection are also well recognized. COVID-19 infection is specifically associated with an increased burden of chronic pain (a) as the consequence of a post-viral syndrome or as the result of viral-associated organ damage; (b) worsening chronic pain due to exacerbation of pre-existing physical or mental complaints; or (c) even generating chronic pain in the absence of infection, at times due to stressors and limitations associated with pandemic restrictions and exacerbation of risk factors such as poor sleep, inactivity, fear, anxiety, and depression [4]. A recent Canadian study emphasized the negative impact of the COVID-19 pandemic on access to pain relief [5].

Early studies and anecdotal information from patients, medical colleagues, and social media suggest that patients may present to chronic pain clinics with a number of complaints not only after COVID-19 infection but also after receiving COVID-19 vaccines. While serious rare adverse effects after vaccination are reported to government agencies [6], isolated case reports have documented persistent neurological or musculoskeletal and other symptoms after vaccination that do not fall under the definition of serious side effects [7,8,9,10,11,12]. 

A representative community survey (REACT-2) of adults in England (more than 600,000 people) showed that at 12 weeks, 37.7% experienced at least one symptom, falling to 21.6% in round six, which included >97,000 participants [13]. Female sex, increasing age, obesity, smoking, vaping, hospitalization with COVID-19, deprivation, and being a healthcare worker were associated with a higher probability of persistent symptoms and Asian ethnicity with a lower probability. The authors concluded that managing the long-term sequelae of COVID-19 will remain a major challenge for affected individuals and their families and for health services.

A Canadian cross-sectional study in 23,757 adults who were 50+ years old and suffered from COVID-19 infection but were not hospitalized, found that the most common persistent symptoms were fatigue, dry cough, muscle/joint pain, sore throat, headache, and runny nose 1 and 3 months post infection [14]. 

The aim of this case-series is to systematically report on a consecutive number of patients presented to a community-based chronic pain clinic located in the Greater Toronto Area, 45 km north of Toronto, Ontario, Canada, because they had acquired persistent pain and other symptoms after COVID-19 infection or vaccination, in an effort to (a) increase our knowledge about the sequelae of infection or vaccination primarily in non hospitalized community dwellers who seek care in the community, and (b) suggest appropriate management.

## 2. Methods

### 2.1. Setting/Population

This is a retrospective descriptive case series derived from a cohort of patients referred and/or treated in a community-based pain clinic with persistent pain and other symptoms post-COVID-19 infection or vaccination, over a period of 4 months (January to April 2022). All patients referred to the clinic fill in a routine intake information sheet after an informed and detailed consent (outlining the use of their data anonymously in aggregate format for research purposes). During the study period (January to April 2022), all new patients referred to the clinic were additionally asked to complete a brief questionnaire regarding development of symptoms after COVID-19 infection or vaccination as part of the usual process of care. Those who responded to the affirmative underwent a further detailed interview. Furthermore, patients currently treated in our clinic for their pain who had developed additional symptoms after COVID-19 infection or vaccination within this 4-month window period were also entered in this study and completed a further detailed interview. The study was approved for retrospective analysis by the University of Toronto Research Ethics Board (Protocol Approval #00042843). 

### 2.2. Data Collection

Information on sex, gender, and age is collected routinely at the time of original consultation for all patients seen in the clinic. For the case series, additional data were extracted from clinical charts and referring physicians’ records, as well as patients’ vaccination passports (granted by the province of Ontario, Canada), regarding details of vaccination (type of vaccine and booster if applicable), description of protracted pain and other symptoms after COVID-19 infection or vaccination, as well as clinical examination findings. 

## 3. Results

The symptomatic cohort with protracted symptoms consisted of 21 patients (17 females and 4 males; F/M 4.25/1) with an age range of 22–79 and a mean age of 46.3 years. Eighteen patients (86%) were younger than 65. Twelve patients (57%) had contracted COVID-19 infection (before or after vaccination), but only one patient had required hospitalization. Sixteen patients had received 1–3 doses of an mRNA vaccine (13 Pfizer-BioNTech and 3 Moderna). Eleven patients were new consultations, while the other ten were patients of our pain clinic when they developed novel symptoms post COVID-19 and/or vaccination. Altogether, 9/21 patients reported symptoms after vaccination only. While 12/21 patients reported testing positive for COVID-19, we did not collect exact data of the type of testing they had (PCR or rapid testing). 

Table 1 and Table 2 provide details of patient symptoms, including pre-existing pain and other symptoms (if any) and symptoms at the latest follow up, ranging from 3 months to 30 months.

### Illustrative Case Reports

While basic details of presentation and outcomes are contained in Table 1 and Table 2, we present details of four cases in the group that developed symptoms after vaccination, as they cover the whole spectrum of pathology (biomedical vs. psychiatric or combination).

Patient B4: A 40-year-old female had a low impact car accident in 2019 and developed multiple pain complaints, anxiety (with panic attacks), and depression. She received two Moderna vaccinations (V1 and V2), in 2021, two months apart. A month after V2, she developed shortness of breath and chest pain. She visited the emergency department on two occasions and was diagnosed with panic attacks. A third visit to the emergency department 6 months after V2 resulted in the diagnosis of pericarditis after a positive echo. She was admitted to another hospital shortly after, where multiple investigations were negative except small pericardial effusion, which was considered incidental (opinion differed from the original diagnosis in the emergency department). To this date (a year or more after V1), the patient has persistent minimal pericardial effusion, currently considered by her cardiologist as lacking clinical significance. She continues to suffer from daily panic attacks and has been unable to leave her home for many months due to anxiety. She also continues to be incapacitated by widespread chest wall pain to light palpations, which have spread to the upper extremities (not consistent with costochondritis or any other structural abnormality and with a completely negative bone scan). 

Interpretation: This patient had significant anxiety and mood disorder pre-vaccination. The development of pericarditis led to aggravated anxiety and chest pain, but she was dismissed twice in the emergency department. When the underlying pericardial effusion decreased substantially, the patient continued with widespread chest and arm pain (consistent with central sensitization), while her anxiety remains treatment-resistant.

Patient B6: A 39 y.o. female received a single Pfizer-BioTech vaccine (V1). The history as obtained through the patient’s narrative is as follows: Within a day after V1, she began experiencing shortness of breath, fatigue, cough, fever, myalgias, body weakness, and pain in the left chest and ribs. A month later she was diagnosed by a cardiologist with pericarditis (on clinical grounds), but this was not confirmed by a subsequent normal echo and stress test. Naproxen and colchicine improved pain by 20% after one week. Symptoms waxed/waned until 3 months after V1, at which time, she started complaining of paresthesia and right leg weakness, as well as “electrical shocks” in her upper back. She stopped colchicine (which she read could produce the above side effects) and within one week all the above symptoms resolved, except her chest pain that not only persisted but also worsened. Four months after V1, the left chest pain spread to her right side. A crash course of prednisone for 5 weeks starting from 20 mg daily with gradual tapering resulted in a 50–60% reduction in her chest and rib pain. Five and a half months post V1, her original symptoms gradually recurred. Seven months after V1, she developed a rash on the right rib cage and was immediately prescribed valacyclovir for a presumed diagnosis of shingles. The rash cleared within 3 days and her pain improved by 50–60%, but only for the 3 days she was on the medication. Since the onset of her pain, she had seen cardiologists, rheumatologists (negative for auto-immune conditions), and other specialists and received chiropractic treatment, physiotherapy, and massage with no benefit. She was seen in our centre for the first time eight months after V1. Palpation revealed tenderness in her lower costochondral joints, both axillae, and across the whole ribcage below the fifth or sixth rib level. A bone scan was ordered. Two months later, the patient had not gone for her bone scan (afraid of the injection). Instead, she informed us she had joined an American blog of “hundreds of patients” all of which had developed symptoms after vaccination and was convinced she had developed Mast Cell Activation Syndrome (MACS). The term refers to an increasingly used diagnosis by individuals who present with multisystem signs and symptoms varying from flushing to hives, abdominal pain, diarrhea, paresthesia, and/or cognitive dysfunction. She asked for further lengthy explanations of the bone scan stating she wanted to have the test, and the test was reordered. She appeared again several weeks later (bone scan again not done), this time having diagnosed herself with costochondritis. Furthermore, she had contacted an American “Centre for COVID-19 Symptoms” and was given a report that indicated (based on her symptoms), that she had ongoing inflammation and suggested a prescription of certain medications including a course of prednisone. However, the site declares that the treatments are experimental, they are not approved by FDA, and the patients must have their own physicians prescribing the recommended medications. We did not consent to prescribe these treatments. The patient continues to be highly disabled and seeks variable treatments and consultations from different specialists including several in the USA. 

Interpretation: The patient developed gradually an inordinate amount of diverse symptoms and the conviction that she suffered from different medical conditions, pursuing self-diagnoses and variable treatments, while she remains highly disabled with numerous subjective symptoms but no objective documentation of any biomedical abnormalities.

Patient B8: The patient (who works as a manual therapist) received three Pfizer -NBioTech vaccines throughout 2021. The day after the first dose (V1), she developed bilateral thumb/radial sided wrist pain that lasted one month. After the second dose (V2), 3 months later, she took a single tablet of ibuprofen and did not experience any symptoms. However, a day after the third dose/booster (V3; 6 months after V2), she woke up with severe bilateral thumb/wrist pain. While the right-hand symptoms resolved over time, the left-hand symptoms persisted. An MSK ultrasound performed 3 months after V3 showed trace fluid in the left abductor pollicis longus tendon, consistent with tenosynovitis. The patient was seen in our centre 5 months after V3. Her passive left wrist range of motion was quite restricted in extension and ulnar and radial deviation, with pain in the radius in all ranges. Her left thumb flexion was restricted with pain and locking. Palpation revealed significant tenderness over the distal radius and abductor pollicis tendon with visible swelling over the distal radiocarpal joint. After reviewing the available literature, our findings convinced us that the patient had developed oligoarthropathy secondary to the vaccination. A crash course of prednisone starting at 60 mg once a day and tapering by 5 mg every second day resulted in a dramatic response by the second day and pain resolution until the patient reached the daily dose of 15 mg. At that point, the pain partially returned, and she was placed on a second protracted course of prednisone (15 mg once a day with dose reduction of 2.5 mg per day every 4 days until the end of the course), resulting in further (but incomplete) symptom resolution. A recent ultrasound revealed persistent large amounts of fluid in abductor pollicis longus and extensor pollicis brevis, consistent with De Quervain’s as well as fluid within the wrist joint. The patient is currently awaiting a rheumatology consultation.

Interpretation: This patient appears to suffer from rare adverse effects of mRNA vaccination with a development of persistent oligoarthropathy.

Patient B9: A 60.y.o.man with an 11-year history of low back and left leg pain, was well managed with epidural injections initially and conservative management subsequently for several years (baseline average pain intensity 2–4/10). He noted significant aggravation of his symptoms after Moderna V1 vaccine and presented to our centre 4 months later with persistent back and left leg pain rated 5–6/10. He responded very well to transforaminal left L4 and L5 epidural steroid injection with pain reduction back to his pre-vaccine baseline (2–4/10). One month after his epidural, he received V3, and within 12 h, he reported excruciating left sciatica that “kept him bedridden” for two weeks. His pain afterwards returned to baseline, without intervention. An EMG/NCT at that time showed chronic and active left L5 radiculopathy. 

Interpretation: After discussions with his neurologist, we all came to the conclusion that the acute flare up of this patient’s sciatica was likely an immune response to the vaccine.

## 4. Discussion 

Our small pragmatic study conducted during a 4-month period in 2022, found 21 patients with protracted symptoms after COVID-19 infection or vaccination, 11 of which were new referrals to our centre. Our centre serves as a tertiary care pain clinic in the community. Since we see on average 42–45 new pain patients per month, the 11 new patients correspond to about 6–6.5% of our monthly referral load (besides 10 patients who were already treated in our centre for their chronic pains). Except one unvaccinated patient in the earlier phases of the pandemic who contracted COVID-19 and was hospitalized (but not in ICU), ultimately becoming a true “long hauler”, all other patients presented to us during routine pain consultations and had never been hospitalized. Patient B9 alerted us to a possible relationship between the vaccination and certain symptoms when he developed severe sciatica within 12 h of receiving the vaccination. We then held informal discussions with a collaborating community neurologist (personal written communication with Dr. V. Basile on 8 February 2022) who advised us he had seen flare-ups of pre-existing pains as well as novel neurological symptoms after infection or vaccination. This ultimately led us to a systematic approach to patients as demonstrated with this study. 

Our study group displayed a number of musculoskeletal, cardiac, neurological, and psychiatric symptoms after COVID-19 infection, vaccination, or both. With the exception of one patient, most patients had sought attention for their symptoms from their family physicians or local specialists. In most cases, pre-existing pains worsened while some patients developed novel pains and other symptoms. In 9/21 patients, pains improved gradually and spontaneously within 2–5 months post-symptom onset. The remaining patients have continued to be partially or significantly impaired by one or more symptoms. 

None of the patients who became symptomatic after vaccination were considered appropriate for referral to the Vaccine Injury Support Program through the Public Agency of Canada: [vaccineinjurysupport.ca] [15].

We undertook a selective review of the literature in an effort to understand the symptoms and presentations of our study group. It appears that the vast body of knowledge regarding COVID-19 infection has been gained from studies of hospitalized patients during the acute symptom phase, as well as in studies following up hospitalized patients after their discharge. 

The preponderance of females in our study group is striking with a F/M ratio of 4.25/1 as opposed to 1.6/1 in our general population, as per our own recently published study [16]. However, female preponderance is supported by the existing literature in hospitalized patients. A recent study showed that, while females exhibit similar symptoms during the acute phase of the infection, they present with greater post-COVID-19 symptoms than males, including higher depressive levels and poor sleep quality eight months after hospital discharge [17]. Similarly, female prepondernce is supported by a systematic review of symptoms during (a) acute COVID-19 infection pertaining to psychiatric issues/mood and musculoskeletal and respiratory problems, as well as (b) long COVID-19 symptoms relating to psychiatric/mood, neurological, dermatological, and other disorders [7].

Based on information from patients hospitalized with COVID-19, a wide spectrum of persistent neurological, musculoskeletal, and psychiatric symptoms has been reported [17,18]. Different painful symptoms (myalgias, arthralgias, abdominal pain, headache, and chest pain) are part of “long haulers” presentations [19]. After discharge from the hospital, the most commonly affected areas by musculoskeletal pain are the lower limbs and lumbar spine, while a large proportion of patients reported also non-specific musculoskeletal pain [20]. Finally, regarding the neurological sequelae after hospitalization for COVID-19, both central and peripheral nervous system involvement has been reported with confusion, headache, and dizziness, as well as anosmia, ageusia, and nerve pain [21,22]. Many of the symptoms described by our patients are indeed part of the long haulers’ spectrum. Studies on community dwellers who had contracted COVID-19 but were never hospitalized demonstrated persistent multisystem symptoms months after infection [13,14].

The search for literature on adverse effects and protracted symptoms after vaccination (particularly following mRNA-based vaccines such as Pfizer -NBioTech and Moderna which have been extensively used in Canada) led us to a couple of interesting studies that explain the worsening symptoms of our patients with pre-existing rheumatological disorders or the symptoms of oligoarthropathy developed by one patient who responded very well, though not completely, to oral steroids (case B8. Connoly et al. [23] published a survey of 1377 participants with rheumatic diseases with 11% of the respondents reporting flare-ups requiring treatment following injection of mRNA-based vaccines. Recently, Ursini et al. [24] published a case series comprising 66 individual patients from 16 different rheumatology centres, with 59% of the patients having received the BNT162b2 (Pfizer) vaccine. The average delay between the day of the ‘trigger’ injection and arthritis onset was 11–13 days. Interestingly, 44.4% of the patients developed symptoms after the first dose. The most common presentation was a polymyalgia rheumatica (PMR)-like picture, followed by oligoarthritis and polyarthritis, with female prevalence 55.6%, 76.2%, and 63%, respectively. Most patients were treated with glucocorticoids, non-steroidal anti-inflammatory drugs, or analgesics, while disease-modifying antirheumatic drugs were used in 28% of the patients with polyarthritis, 24% patients with oligoarthritis, and 11% of patients with PMR-like presentation. Three quarters of the latter group achieved full remission of symptoms after 2 weeks, while 67% of patients with polyarthritis had an active disease after an average follow-up of 6 weeks. The authors concluded that these inflammatory complications are quite uncommon and “probably reflect a transient reactogenic response to the vaccine rather than a structured, chronic inflammatory joint disease”. 

As for the possibility of pericarditis post-mRNA vaccination, this is an extremely rare occurrence; a meta-analysis of 22 studies actually reported that the rate of myopericarditis is no higher after COVID-19 vaccination as after other common vaccinations, and that it may even be lower in some cases [25].

In terms of neurological symptoms and signs post vaccination, isolated case reports have presented cases of biopsy-proven small fiber neuropathy [26] or disabling and variable symptoms ranging from isolated and bilateral scintillating scotoma to unspecific multisystem manifestations [11,27].

Finally, the worsening of anxiety and mood disorders, observed in several of our patients, has been reported in several studies post-COVID-19 hospitalization, particularly in women as we reported earlier [17,19]. Psychiatric symptoms seemed to be disabling and protracted in a minority of our patients (primarily in the group that acquired symptoms after vaccination), while others responded well to the appropriate pharmacotherapy for depression, anxiety and pain, and/or interdisciplinary pain management.

## 5. Study Limitations

This is a small descriptive study presenting a case series seen within a 4-month period in a community pain clinic in the Greater Toronto Area. The sample is too small to allow for the generalizability of the results and significantly add to the body of knowledge regarding long-term sequelae of COVID-19 infection and adverse effects of mRNA vaccines. Confirmation of COVID-19 infection was obtained through the referring physician’s referral letter, and we had no direct access to the method of confirmation of the infection (PCR or rapid test).

## 6. Conclusions

Our small case series provides information related to long-term sequelae of COVID-19 infection and adverse effects of mRNA vaccines in community dwellers who did not require hospitalization (except one). Extrapolating the literature from hospitalized patients during the acute phase of infection and after discharge from the hospital, as well as literature reporting on rare adverse effects post vaccination, our small case series provides a “bird’s eye view” of multiple systemic, musculoskeletal, neurological, and psychiatric symptoms after COVID-19 infection or vaccination in patients who were not hospitalized, despite the limitations of the study as cited above. Such patients with variable presentation are visiting family practices, neurological and rheumatological services, psychiatric clinics, pain clinics, and emergency departments and run the risk of been dismissed if the health professionals lack the appropriate knowledge. 

More extensive research is needed to confirm our findings in the general community practices and how to educate health professionals to properly identify and treat such patients.

## Figures and Tables

**Table 1:** Demographics and symptoms of study patients after COVID-19 infection.

Patients	Sex/Age	Pre-Existing Symptoms	Persistent Symptoms Post-COVID-19 Infection	Follow up Post Infection	Outcome as of Last Visit on File after Infection
A1	F/48	Back pain as of November 2020	First infection led to persistent fatigue, brain fog. Second infection 3 months later resulted in increased fatigue, brain fog, night cough, and increased back pain	6 months	Recovered to baseline, remains at work full time, physically active
A2	F/49	Neck, right shoulder pain since 2017	Worsening of neck pain since infection	3 months	Persistent neck pain worse than before infection
A3	F/32	Mid back/right arm pain since September 2019	Post infection worsening of mid back pain plus fatigue, brain fog, body weakness, nausea, tinnitus, tachycardia	5 months	Most symptoms cleared by 5 months and pain improved more than baseline
A4	F/56	Fibromyalgia since 2015. Did well with pain management * program by September 2019	Infection a year later required hospitalization (but not ICU), much worse body pain, shortness of breath, cough, fatigue, brain fog, depression, weight loss, sleep disorder, parosmia, peripheral neuropathy	30 months	Severely disabled with multi-system symptoms, out of control body pain, and severe depression (typical “long hauler”)
A5	M/29		Two weeks after 10-day isolation period post infection resulted in severe sciatica with negative spinal MRI	4 months	Persistent back pain and radicular symptoms and signs
A6	F/65		After infection chest pain, fever, cough, body aches, limb paresthesia, hair loss, fatigue, loss of taste and smell for 7 months, and bladder urgency and frequency further worsened post vaccination, a year after original infection.	30 months	All symptoms improved with duloxetine and pain management. Bladder symptoms improved at 30 months post infection
A7	F/70	Hand pains due to psoriatic arthritis well controlled with disease modifiers	Severe hand symptoms since infection plus significant fatigue	7 months	Hand pain improved but has not returned to baseline while fatigue continues
A8	F/56	Long standing rheumatoid arthritis with polyarticular pain on disease modifiers	Much worse polyarticular pain post infection with additional myalgias, fever, cough, and depression	5 months	All symptoms improved substantially by last follow up (mood and pain aided by duloxetine)
A9	F/61	22-year history of right ankle and left knee arthritis, and CRPS ** right leg, all in good control	Novel total body pain and skin sensitivity to touch (allodynia) post infection	7 months	Partial only improvement of diffuse body pain and allodynia
A10	F/44	Chronic neck and upper quadrant pain, much improved with pain management	Persistent worsening of pains post infection	7 months	Severely decompensated after infection
A11	F/30	Pre-existing anxiety and migraines	Worsening of anxiety and migraines plus novel neck/arm pains post infection	10 months	Much improved with pain management program
A12	M/42	Chronic neck pain and anxiety	Worsening of anxiety and musculoskeletal pains post infection	1.5 months	Improved with pain management

Three patients received Moderna (indicated in text) and the remainder Pfizer-NBioTech. V1, V2, and V3 correspond to first, second, and third (booster) vaccine, respectively. * Pain management included exercise therapy, nutritional counselling, cognitive behavioural therapy, and mindfulness. ** CRPS = Complex Regional Pain Syndrome.

**Table 2:** Demographics and symptoms of study patients after COVID-19 vaccination.

Patients	Sex/Age	Pre-Existing Symptoms	Persistent Symptoms Post-COVID-19 Vaccination	Follow up Post COVID-19 Vaccination	Outcome as of Last Visit on File after COVID-19 Vaccination
B1	F/37	Neck and trapezius pain	Worse pains within 24 hrs after V2; lymphadenopathy and new left pectoralis pain after V3	15 months post V3	Persistent pains (neck, trapezius, left pectoralis) with little response to medications and manual therapy
B2	F/79	Pre-existing multisite pains over 40 years	Pre-existing pains much worse within a day after V1 plus diffuse burning distal leg pains	18 months after vaccination	Persistent (old and new) pains
B3	F/25	Chronic low back pain	Pre-existing pain worsened 2 weeks after V2	18 months after V2	Persistent low back pain worse than pre-vaccination
B4	F/39	Multisite pre-existing pains, anxiety, and depression after a low impact car accident	Severe worsening of pre-existing pains, anxiety, and depression, plus pericarditis after V1 and V2 (Moderna) with diffuse chest pain and numerous panic attacks	30 months after V2	Unremitting symptoms (see case report below)
B5	M/22		Disabling chest pain 2 weeks after V1 Moderna (cardiac investigations negative	10 months after V1	Persistent chest pain with negative phys. examination. Offered pain management but declined
B6	F/39		Developed deep ache left chest, axilla, shortness of breath, and fever, after V1	12 months after V1	Highly symptomatic a year later (see case report below)
B7	F/56		3-year history of undiagnosed left flank pain, which worsened a year later after V1		Seen a year after V1. On examination, clear evidence of left thoracic root irritation at T7-8
B8	F/33		Wrist and thumb inflammation one month after V1; persistent oligoarthropathy after V3	5 months after V3	Objective and subjective inflammatory signs markedly improved on crash course of prednisone (see case report below)
B9	M/60	11-year history of low back pain in good control	Significant aggravation of low back pain after V1 (Moderna); acute sciatica 12 hrs after V3		Pain returned to baseline, 2 weeks after V3 (see case report below)

Three patients received Moderna (indicated in text) and the remainder Pfizer-NBioTech. V1, V2, and V3 correspond to first, second, and third (booster) vaccine, respectively.

## Data Availability

Data will not be available in a public repository due to patient privacy and confidentiality issues.
